# Incidence of breast cancer attributable to breast density, modifiable and non-modifiable breast cancer risk factors in Singapore

**DOI:** 10.1038/s41598-019-57341-7

**Published:** 2020-01-16

**Authors:** Peh Joo Ho, Hannah Si Hui Lau, Weang Kee Ho, Fuh Yong Wong, Qian Yang, Ken Wei Tan, Min-Han Tan, Wen Yee Chay, Kee Seng Chia, Mikael Hartman, Jingmei Li

**Affiliations:** 1 Genome Institute of Singapore, 60 Biopolis Street, Genome, #02-01, Singapore, 138672 Singapore; 2 Saw Swee Hock School of Public Health, National University of Singapore and National University Health System, Singapore, Singapore; 3 Faculty of Science, National University of Singapore, Singapore, Singapore; 4 Department of Applied Mathematics, Faculty of Engineering, University of Nottingham Malaysia, Selangor, Malaysia; 5 Cancer Research Malaysia, 1 Jalan SS12/1A, Subang Jaya, 47500 Selangor, Malaysia; 6 National Cancer Centre Singapore, Singapore, Singapore; 7 Institute of Bioengineering and Nanotechnology, Singapore, Singapore; 8 Department of Surgery, Yong Loo Lin School of Medicine National University of Singapore, Singapore, Singapore

**Keywords:** Epidemiology, Breast cancer

## Abstract

Incidence of breast cancer is rising rapidly in Asia. Some breast cancer risk factors are modifiable. We examined the impact of known breast cancer risk factors, including body mass index (BMI), reproductive and hormonal risk factors, and breast density on the incidence of breast cancer, in Singapore. The study population was a population-based prospective trial of screening mammography - Singapore Breast Cancer Screening Project. Population attributable risk and absolute risks of breast cancer due to various risk factors were calculated. Among 28,130 women, 474 women (1.7%) developed breast cancer. The population attributable risk was highest for ethnicity (49.4%) and lowest for family history of breast cancer (3.8%). The proportion of breast cancers that is attributable to modifiable risk factor BMI was 16.2%. The proportion of breast cancers that is attributable to reproductive risk factors were low; 9.2% for age at menarche and 4.2% for number of live births. Up to 45.9% of all breast cancers could be avoided if all women had breast density <12% and BMI <25 kg/m^2^. Notably, sixty percent of women with the lowest risk based on non-modifiable risk factors will never reach the risk level recommended for mammography screening. A combination of easily assessable breast cancer risk factors can help to identify women at high risk of developing breast cancer for targeted screening. A large number of high-risk women could benefit from risk-reduction and risk stratification strategies.

## Introduction

Breast cancer is the most common cancer among women, accounting for 25% of all female cancers globally^1^. In particular, breast cancer incidence in Asia is rising more rapidly than in the West^2^. An annual increase of 3.9% was reported in Singapore, compared to 1.5% in the United States^3^. Multiple risk factors for breast cancer are well-established; these include breast density, reproductive (parity and age at first birth), menstrual (menopausal status and age at menarche), and modifiable lifestyle factors such as body mass index (BMI), hormone replacement therapy (HRT) use and alcohol consumption^4–6^. Quantifying the proportion of breast cancer cases that can be attributed to each of these risk factors can aid in developing population-specific public health interventions and strategies to reduce breast cancer incidence.

The population attributable risk (PAR) is the proportion of disease cases that would not have occurred if the risk factor was removed from the population^7^. The PAR of any risk factor is dependent on the magnitude of the association between the risk factor and the disease, and the prevalence of the risk factor in the population^8,9^. The prevalence of risk factors may vary among different populations and can be influenced by lifestyle preferences, cultural habits, environmental factors and socio-economic status. PAR estimates may thus vary across different populations and at different time periods even if the association between the risk factor and disease does not change^9^.

Previous studies on PAR of breast cancer risk factors have mostly been conducted in Western populations in the United States^8–12^, Europe^13–15^ and Australia^16^. Reported PARs of modifiable lifestyle risk factors ranged from 1% for alcohol consumption (relative risk of 1.4 was used)^10^ to 39.3% for breast density (relative risks of 1.57 and 1.84 were used) assessed using the Breast Imaging Reporting and Data System (BI-RADS)^8^. In addition, PAR of the same risk factor can have a wide range depending on the population studied (e.g. 2%^10^ to 19.4%^13^ for HRT use).

Southeast Asia is a geographically expansive and populous region amounting to ~656 million people in 2018, equivalent to ~8.6% of the world’s population^17^. The region is characterized by the coexistence of various cultures and ethnic groups. Thus far, the PAR of breast cancer attributed to breast cancer risk factors have been studied mainly in East Asian populations (e.g. Korea^18^ and mainland China^19,20^). Singapore is a multiracial and multicultural country comprising of three main Asian populations, with ethnic Chinese (74.3% of the citizen population), Malays (13.4%), and Indians (9.1%) making up the majority of the population^21^. In this study, we examined the impact of known breast cancer risk factors, including BMI, reproductive and hormonal risk factors, and breast density on the incidence of breast cancer.

## Methods

### Study population

**Figure 1:**
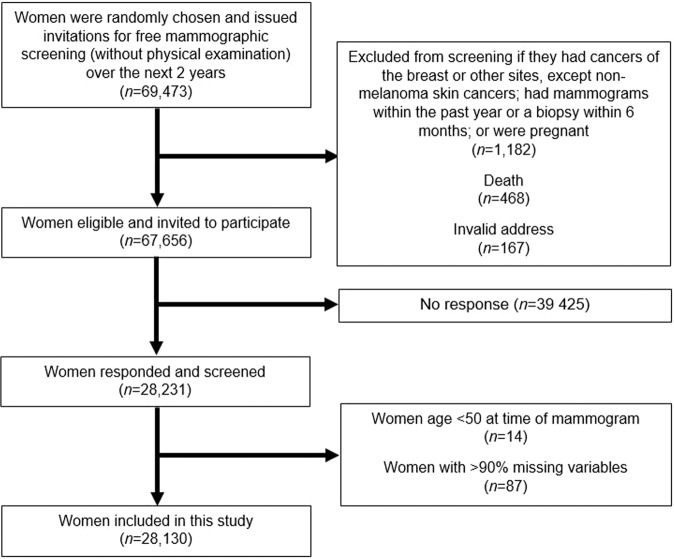
Flow diagram of how analytical cohort was derived.

The Singapore Breast Cancer Screening Project (SBCSP) was a population-based prospective trial of screening mammography in Singapore. In this project, 69,473 women aged 50–64 years were randomly selected and invited for a single two-view mammogram examination from 1994 through 1997^22^. Further details of this program have been previously described^22^. In brief, women were excluded if they had cancers of the breast or other sites (except non-melanoma skin cancer), had mammography done or breast biopsy in the past one year prior to screening, or were pregnant (*n* = 1,182). Further exclusions were made due to death (*n* = 468) or invalid address (*n* = 167). Of the eligible 67,656 women, 41.7% (*n* = 28,231) participated and were screened as part of SBCSP (see flow diagram in Fig. 1). The concern of the low participation rates was addressed in a previous publication, in brief, the incidence of breast cancer in non-respondents was slightly less than that of women not invited for screening (P = 0.03), however the breast cancer stage distribution did not differ significantly^22^. We further excluded 101 (0.4%) of participants; which included 14 (13.9%) participants with reported age <50 years and 87 (86.1%) participants with missing details on intended study variables. Ethical approval and the waiver of the need for informed consent was approved by SingHealth Centralised Institutional Review Board (REF: 205-001). In addition, this study used existing anonymous data. All research was performed in accordance with relevant regulations.

### Identification of breast cancer cases

Incident cases of invasive and non-invasive breast cancer cases diagnosed after study entry (date of screen) until December 2007 (the end of study), were identified through record linkage with the Singapore Cancer Registry using unique national registration identity (card) numbers. The population-based national registry records all cases of cancer diagnosed in Singapore, with an overall completeness of 98.1%^23^.

### Variables of interest

The participants of SBCSP completed a questionnaire, prior to receiving their mammogram screening results, detailing their demographics and lifestyle factors on the day they attended screening, which included modifiable risk factors, BMI (kg/m^2^: <18.5, 18.5–24.9, 25.0–29.9, 30.0–34.9, ≥35.0^24^) and ever smoke (yes, no). Non-modifiable risk factors studied included age at study entry (years, 50–59, 60–64), ethnicity (Chinese, Malay, Indian, other), family history of breast cancer (yes, no), and personal history of benign breast disease (yes, no). Reproductive and hormonal characteristics included menopausal status (pre-menopausal, post-menopausal), age at menarche (years, ≤13, 14–15, ≥16), number of live births (0, 1–2, 3–4, ≥5), age at first live birth (age ≤30, age >30), breastfeeding history (yes, no), ever used contraceptive (yes, no) and ever used hormone replacement therapy (yes, no). Breast density in the form of percent mammographic density was calculated from screen-film mammography images originally collected during the SBCSP which had been digitalized between February 2012 and February 2013. Breast density was categorized into quartiles, with values of 12.29%, 18.24% and 26.03% corresponding to the first quartile, median and third quartile respectively. A fully-automated thresholding method was used to measure breast density as described previously^25,26^.

### Statistical analysis

Chi-square tests were used to test for significant associations between each risk factor and incidence of breast cancer.

PAR estimates the proportion of disease cases that could be prevented if everyone in the population were shifted to the reference category. Here, the PAR for each risk factor was estimated using the method described by Bruzzi *et al*., by assuming a case-control study design^7^. This method allows for multivariable adjusted relative risk estimates and requires only the distribution of the risk factors among the case subjects. The PAR was computed using the formula:

$$1-\sum _{j}\frac{p{d}_{j}}{R{R}_{j}}$$

where ${{pd}}_{j}$ is the proportion of all cases in stratum $j$ of the risk factor and ${{RR}}_{j}$ is the univariable/ multivariable adjusted relative risk associated with that stratum^9^.

The PAR is useful in estimating how much of the disease burden in the population could be reduced if certain risk factors were eliminated^27^. Individual risk factors may interact in their contributions to overall breast cancer risk. Consequently, PARs for individual risk factors often overlap and the sum may exceed 100%^28^. It is noteworthy that there are several underlying assumptions in the interpretation of PAR. The PAR assumes that the risk factor is causal rather than merely associated with the disease. The PAR also assumes that the elimination of the risk factor does not affect the distribution of other risk factors, which is unlikely to always hold true^16^. In addition, the PAR is sensitive to the reference category chosen and distribution of risk factors in the population - caution needs to be taken when comparing our results to other studies or different time periods^8,10,11^. Where the reference category was not the lowest risk of breast cancer, PAR was estimated by shifting only women in higher risk category to the reference category. For example, in the estimation of the PAR for number of live births, the reference level was 1–2. The reported PAR will then be on nulliparous women (highest risk category) compared to the reference level.

The relative risk (${{RR}}_{j}$, where $j$ = 1, …, maximum number of strata of the variable of interest) used in the estimation of PAR were estimated using the odds ratios obtained from both univariable/multivariable logistic regression models (age, BMI, age at menarche, and breast density were adjusted as continuous variables, unless they were the risk factor which PAR was estimated, in addition to smoking status, ethnicity, family history, personal history of benign breast disease, number of live births, HRT use), based on the actual dataset. Missing values for continuous variables were replaced by the mean of the variable during adjustment. Bootstrapping using 2,000 iterations, with the estimated ${RR}$, was used to estimate the 95% confidence interval of PAR.

Pair-wise combinations of risk factors were studied. The reference category was taken to be the reference risk categories of both risk factors studied – for example, the combination of BMI (reference level = 18.5–24.9) and family history (reference level = no) would have women with BMI of 18.5–24.9 and without family history as the reference category. Similar to the analysis of one risk factor, PAR was estimated by shifting only women in higher risk category to the reference category.

The age-specific absolute risk of developing breast cancer in each risk categories was calculated under the assumption that the average age-specific breast cancer incidence over all risk categories agreed with the population breast cancer incidence. The details of the method have been previously described^29^. Projection of absolute risk distribution was based on the breast cancer incidence rates^30^ and mortality rates in Singapore^29,31^. A random selection of 50% of study participants was used to build the logistic model to categorize women with differing risks of breast cancer (<30, 30–60, 60–90, ≥90^th^ percentiles) based on non-modifiable risk factors (ethnicity, family history of breast cancer, history of benign breast disease, parity, age at menarche, number of live births and age at first live birth). The relative and absolute risks of developing breast cancer by percentiles of non-modifiable risk factors distribution were calculated using the remaining 50% of study participants. Bootstrapping using 2,000 iterations was done to estimate the odds ratios used in the projection of absolute risks. Absolute risk was computed only for non-modifiable risk factors as the values do not change over a woman’s lifetime. For comparison, we added BMI, smoking status and breast density to the risk model and estimated the absolute risk based on the new risk categorization of women. The discriminatory accuracy of non-modifiable risk factors on predicting breast cancer risk was evaluated using the area under the receiving operating curve (AUC), estimated using the *pROC* package in R^32^. The median and interquartile range of AUC from the bootstrapping method was reported. All statistical analyses were performed using R (version 3.4.3).

### Ethics approval and consent to participate

This study uses existing anonymous data from the Singapore Breast Cancer Screening Project. The original study received ethics approval from SingHealth Centralised Institutional Review Board (REF: 205-001).

### Consent for publication

All authors approved the manuscript and consented to its publication.

## Results

A total of 28,130 women were included in this study, of whom 474 (1.7%) developed breast cancer. The study population was predominantly ethnic Chinese (84.2%) with smaller percentages of Malays (5.6%), ethnic Indians (5.0%) and other races (5.2%) (Table 1). The majority of women had at least one biological child (92.7%) and had previously breastfed (63.8%). Majority also reported no contraceptive use (61.8%) and no HRT use (86.7%). Nine in ten women (89.6%) were post-menopausal (the description of post-menopausal women by breast cancer occurrence is presented in Table 1). Further details describing the study population may be found in Supplementary Tables 1–3.

**Table 1:** Distribution of modifiable, non-modifiable, reproductive and hormonal risk factors, and breast density in 28,130 Asian women, of which 25,207 were post-menopausal at the time of screening.

	Breast cancer in all women			Breast cancer in post-menopausal women		
	No, *n* = 27,656 (98.3%)	Yes, *n* = 474 (1.7%)	P-value	No, *n* = 24,793 (98.4%)	Yes, *n* = 414 (1.6%)	P-value
**Modifiable**						
**Body mass index group (kg/m**^**2**^**)**						
<18.5	14,400 (52.1)	235 (49.6)	0.336	12,824 (51.7)	199 (48.1)	0.339
18.5–24.9	1,256 (4.5)	15 (3.2)		1,166 (4.7)	15 (3.6)	
25.0–29.9	9,199 (33.3)	169 (35.7)		8,270 (33.4)	151 (36.5)	
30.0–34.9	2,294 (8.3)	46 (9.7)		2,067 (8.3)	41 (9.9)	
≥35.0	496 (1.8)	9 (1.9)		455 (1.8)	8 (1.9)	
Unknown	11 (0.0)	0 (0.0)		11 (0.0)	0 (0.0)	
**Smoke (Ever)**						
No	25,934 (93.8)	451 (95.1)	0.249	23,164 (93.4)	392 (94.7)	0.366
Yes	1,722 (6.2)	23 (4.9)		1,629 (6.6)	22 (5.3)	
**Non-modifiable**						
**Age group, years**						
50–59	18,545 (67.1)	342 (72.2)	0.020	15,710 (63.4)	282 (68.1)	0.050
≥60	9,111 (32.9)	132 (27.8)		9,083 (36.6)	132 (31.9)	
**Ethnicity**						
Chinese	23,273 (84.2)	409 (86.3)	0.029	20,801 (83.9)	356 (86.0)	0.020
Malay	1,569 (5.7)	13 (2.7)		1,444 (5.8)	11 (2.7)	
Indian	1,369 (5.0)	27 (5.7)		1,239 (5)	26 (6.3)	
Other	1,445 (5.2)	25 (5.3)		1,309 (5.3)	21 (5.1)	
**Family history of breast cancer**						
No	26,635 (96.3)	439 (92.6)	<0.001	23,903 (96.4)	382 (92.3)	<0.001
Yes	696 (2.5)	32 (6.8)		601 (2.4)	30 (7.2)	
Unknown	325 (1.2)	3 (0.6)		289 (1.2)	2 (0.5)	
**History of benign breast disease**						
No	26,228 (94.8)	424 (89.5)	<0.001	23,559 (95.0)	372 (89.9)	<0.001
Yes	1,428 (5.2)	50 (10.5)		1,234 (5.0)	42 (10.1)	
**Reproductive and hormonal**						
**Age at menarche, years**						
≤13	9,659 (34.9)	209 (44.1)	<0.001	8,488 (34.3)	179 (43.2)	<0.001
14–15	10,381 (37.6)	167 (35.2)		9,302 (37.5)	143 (34.5)	
≥16	7,604 (27.5)	98 (20.7)		6,991 (28.2)	92 (22.2)	
**Menopausal status**						
Pre	2,863 (10.4)	60 (12.7)	0.120	—		
Post	24,793 (89.6)	414 (87.3)				
**Number of live births**						
0	1,980 (7.2)	65 (13.7)	<0.001	1,782 (7.2)	58 (14.0)	<0.001
1-2	4,282 (15.5)	92 (19.4)		3,686 (14.9)	81 (19.6)	
3-4	9,448 (34.2)	179 (37.8)		8,185 (33.0)	149 (36.0)	
≥5	11,946 (43.2)	138 (29.1)		11,140 (44.9)	126 (30.4)	
**Age at first live birth**^**a**^						
Age ≤30 years	23,467 (91.4)	358 (87.5)	0.008	21,050 (91.5)	312 (87.6)	0.013
Age >30 years	2,209 (8.6)	51 (12.5)		1,961 (8.5)	44 (12.4)	
**Ever breast feed**^**a**^						
No	7,810 (30.4)	161 (39.4)	<0.001	6,886 (29.9)	141 (39.6)	<0.001
Yes	17,692 (68.9)	245 (59.9)		15,972 (69.4)	213 (59.8)	
Unknown	174 (0.7)	3 (0.7)		153 (0.7)	2 (0.6)	
**Contraceptive**						
No	17,072 (61.7)	311 (65.6)	0.086	15,620 (63)	283 (68.4)	0.027
Yes	10,584 (38.3)	163 (34.4)		9,173 (37)	131 (31.6)	
**Hormone replacement therapy use**						
No	23,996 (86.8)	382 (80.6)	<0.001	21,712 (87.6)	339 (81.9)	<0.001
Yes	3,660 (13.2)	92 (19.4)		3,081 (12.4)	75 (18.1)	
**Others**						
**Percentage breast density, quartiles**						
0–12.29	5,983 (21.6)	53 (11.2)	<0.001	5,798 (23.4)	53 (12.8)	<0.001
12.30–18.24	5,983 (21.6)	81 (17.1)		5,578 (22.5)	75 (18.1)	
18.25–26.03	5,982 (21.6)	124 (26.2)		5,295 (21.4)	111 (26.8)	
26.04–100	5,983 (21.6)	176 (37.1)		4,743 (19.1)	142 (34.3)	
Unknown	3,725 (13.5)	40 (8.4)		3,379 (13.6)	33 (8.0)	

^a^Excludes women who are nulliparous.

Non-Malay ethnicity, family history of breast cancer, personal history of benign breast disease, younger age at menarche, fewer number of live births, and higher breast density were associated with increased risks of developing breast cancer (Table 2), which persisted after adjustment. BMI was not associated with breast cancer risk in the univariate model, but after adjusting for other variables, higher BMI was found to be associated with increased breast cancer risk. Results are discussed separately under sub-sections on modifiable, non-modifiable, reproductive and hormonal risk factors, and breast density below.

**Table 2:** Population attributable risk proportion of known risk factors in 28,130 Asian women of which 25,207 were post-menopausal. The association of known risk factors and breast cancer was estimated using logistic regression.

Characteristics	Breast cancer in all women (*n* = 28,130)				Breast cancer in post-menopausal women (*n* = 25,207)			
	Univariable		Multivariable^a^		Univariable		Multivariable^a^	
	OR (95% CI)	PAR (95% CI)	OR (95% CI)	PAR (95% CI)	OR (95% CI)	PAR (95% CI)	OR (95% CI)	PAR (95% CI)
**Modifiable**								
**BMI (kg/m**^**2**^**)**								
18.5–24.9	1.00 (Reference)	—	1.00 (Reference)	16.2 (14.5 to 17.4)	1.00 (Reference)	—	1.00 (Reference)	17.7 (15.9 to 19.1)
<18.5	0.73 (0.43 to 1.24)		0.54 (0.32 to 0.92)		0.83 (0.49 to 1.41)		0.60 (0.35 to 1.03)	
25.0–29.9	1.13 (0.92 to 1.38)		1.45 (1.18 to 1.78)		1.18 (0.95 to 1.46)		1.51 (1.21 to 1.88)	
30.0–34.9	1.23 (0.89 to 1.69)		1.82 (1.30 to 2.55)		1.28 (0.91 to 1.80)		1.89 (1.32 to 2.70)	
≥35.0	1.11 (0.57 to 2.18)		1.73 (0.87 to 3.44)		1.13 (0.56 to 2.31)		1.73 (0.83 to 3.59)	
**Smoke**								
No	1.00 (Reference)	—	1.00 (Reference)	—	1.00 (Reference)	—	1.00 (Reference)	—
Yes	0.77 (0.50 to 1.17)		0.91 (0.59 to 1.39)		0.80 (0.52 to 1.23)		0.91 (0.59 to 1.39)	
**Non-modifiable**								
**Age group, years**								
50-69	1.00 (Reference)	—	1.00 (Reference)	—	1.00 (Reference)	—	1.00 (Reference)	—
≥70	0.79 (0.64 to 0.96)		1.12 (0.90 to 1.39)		0.81 (0.66 to 1.00)		1.11 (0.89 to 1.38)	
**Ethnicity**								
Malay	1.00 (Reference)	51.7 (50.8 to 52.3)	1.00 (Reference)	49.4 (48.6 to 50.0)	1.00 (Reference)	54.4 (53.5 to 55.0)	1.00 (Reference)	52.5 (51.6 to 53.1)
Chinese	2.12 (1.22 to 3.69)		2.06 (1.17 to 3.62)		2.25 (1.23 to 4.10)		2.19 (1.19 to 4.06)	
Indian	2.38 (1.22 to 4.63)		1.95 (1.00 to 3.81)		2.75 (1.36 to 5.60)		2.25 (1.10 to 4.58)	
Other	2.09 (1.06 to 4.10)		1.77 (0.90 to 3.48)		2.11 (1.01 to 4.38)		1.78 (0.85 to 3.72)	
**Family history of breast cancer**								
No	1.00 (Reference)	4.3 (2.9 to 5.5)	1.00 (Reference)	3.8 (2.6 to 4.8)	1.00 (Reference)	4.9 (3.2 to 6.2)	1.00 (Reference)	4.4 (2.9 to 5.5)
Yes	2.79 (1.93 to 4.03)		2.30 (1.59 to 3.34)		3.12 (2.14 to 4.57)		2.56 (1.74 to 3.77)	
Unknown	0.56 (0.18 to 1.75)		0.48 (0.15 to 1.52)		0.43 (0.11 to 1.75)		0.37 (0.09 to 1.51)	
**History of benign breast disease**								
No	1.00 (Reference)	5.7 (4.2 to 6.8)	1.00 (Reference)	4.3 (3.2 to 5.2)	1.00 (Reference)	5.4 (3.9 to 6.6)	1.00 (Reference)	4.2 (3.0 to 5.0)
Yes	2.17 (1.61 to 2.92)		1.69 (1.25 to 2.29)		2.16 (1.56 to 2.98)		1.69 (1.22 to 2.36)	
**Reproductive and hormonal**								
**Age at menarche, years**^**b**^								
14–15	1.00 (Reference)	11.3 (10.2 to 12.2)	1.00 (Reference)	9.2 (8.2 to 9.8)	1.00 (Reference)	11.7 (10.5 to 12.7)	1.00 (Reference)	9.6 (8.6 to 10.4)
≥16	0.80 (0.62 to 1.03)		0.87 (0.67 to 1.12)		0.86 (0.66 to 1.11)		0.93 (0.71 to 1.21)	
≤13	1.35 (1.10 to 1.65)		1.26 (1.02 to 1.56)		1.37 (1.10 to 1.71)		1.28 (1.02 to 1.61)	
**Number of live births**								
1-2	1.00 (Reference)	4.7 (3.7 to 5.6)	1.00 (Reference)	4.2 (3.3 to 5.0)	1.00 (Reference)	4.7 (3.7 to 5.6)	1.00 (Reference)	—
0	1.53 (1.11 to 2.11)		1.45 (1.04 to 2.00)		1.53 (1.11 to 2.11)		1.39 (0.99 to 1.97)	
3-4	0.88 (0.68 to 1.14)		0.97 (0.75 to 1.26)		0.88 (0.68 to 1.14)		0.92 (0.70 to 1.21)	
≥5	0.54 (0.41 to 0.70)		0.71 (0.53 to 0.93)		0.54 (0.41 to 0.70)		0.68 (0.50 to 0.91)	
**Age at first live birth**^**b**^								
Age ≤30 years	1.00 (Reference)	4.2 (3.2 to 5.0)	1.00 (Reference)	—	1.00 (Reference)	4.2 (3.0 to 5.0)	1.00 (Reference)	—
Age >30 years	1.51 (1.12 to 2.04)		1.15 (0.84 to 1.59)		1.51 (1.10 to 2.08)		1.09 (0.77 to 1.54)	
**Ever breastfed**^**b**^								
Yes	1.00 (Reference)	13.1 (11.5 to 14.3)	1.00 (Reference)	—	1.00 (Reference)	13.8 (12.1 to 15.1)	1.00 (Reference)	—
No	1.49 (1.22 to 1.82)		1.17 (0.94 to 1.46)		1.54 (1.24 to 1.90)		1.19 (0.94 to 1.51)	
Unknown	1.25 (0.39 to 3.93)		0.93 (0.29 to 2.98)		0.98 (0.24 to 3.98)		0.68 (0.16 to 2.81)	
**Contraceptive**								
Yes	1.00 (Reference)	—	1.00 (Reference)	—	1.00 (Reference)	14.5 (13.5 to 15.2)	1.00 (Reference)	—
No	1.18 (0.98 to 1.43)		1.04 (0.85 to 1.28)		1.27 (1.03 to 1.56)		1.10 (0.88 to 1.38)	
**HRT use**								
No	1.00 (Reference)	7.1 (5.8 to 8.1)	1.00 (Reference)	—	1.00 (Reference)	6.5 (5.2 to 7.5)	1.00 (Reference)	—
Yes	1.58 (1.25 to 1.99)		1.25 (0.99 to 1.59)		1.56 (1.21 to 2.01)		1.20 (0.92 to 1.56)	
**Others**								
**Breast density**								
0–12.29	1.00 (Reference)	48.3 (46.2 to 50.0)	1.00 (Reference)	48.6 (46.4 to 50.3)	1.00 (Reference)	45.3 (42.7 to 47.1)	1.00 (Reference)	45.4 (42.8 to 47.3)
12.30–18.24	1.53 (1.08 to 2.16)		1.55 (1.09 to 2.21)		1.47 (1.03 to 2.09)		1.49 (1.04 to 2.14)	
18.25–26.03	2.34 (1.69 to 3.23)		2.42 (1.72 to 3.39)		2.29 (1.65 to 3.19)		2.37 (1.68 to 3.34)	
26.04–100	3.32 (2.44 to 4.52)		3.39 (2.41 to 4.77)		3.28 (2.38 to 4.50)		3.31 (2.33 to 4.70)	
Unknown	1.21 (0.80 to 1.83)		1.13 (0.75 to 1.72)		1.07 (0.69 to 1.65)		0.99 (0.64 to 1.53)	

^a^Adjusted for age, BMI, age at menarche and breast density as continuous variables, in addition to ethnicity, family history, history of benign breast disease, number of live births and HRT use, unless it was the factor which PAR was estimated. ^b^Excludes women who are nulliparous.

PAR: population attributable risk. BMI: Body mass index. HRT: Hormone replacement therapy. OR: Odds ratio. CI: Confidence interval.

Among risk factors significantly associated with breast cancer, the most prevalent risk factor was ethnicity, with 94.4% non-Malays, followed by high breast density (65.2%), BMI ≥25 kg/m^2^ (43.4%), and age at menarche ≤13 (35.1%). Other risk factors were of low prevalence; among all women 7.2% were nulliparous, 5.3% had a personal history of benign breast disease and 2.6% had a family history of breast cancer.

### PARs of modifiable risk factors

Up to 16.2% (95% CI: 14.5–17.4) of breast cancers could be prevented if all women had a BMI of <25 kg/m^2^ (Table 2). Large PAR was observed for the combination of BMI and ethnicity (51.4%, 95% CI: 50.4–52.1) and for BMI and breast density (45.9%, 95% CI: 43.7–47.6), in all women (Table 3).

**Table 3:** Population attributable risk (PAR) for combinations of risk factors. Where the risk factors (as categorical variables) were not studied in combination, the following risk factors were adjusted for breast density (as a continuous variable), body mass index (as a continuous variable), ethnicity, age at recruitment (as a continuous variable), family history of breast cancer, age at menarche (as a continuous variable), age at first live birth, and hormone replacement therapy use.

Risk factor 1	Risk factor 2	Population attributable risk (95% Confidence Interval)^1^	
		All women	Post-menopausal women
Body mass index	Ethnicity	51.4 (50.4 to 52.1)	40.1 (39.1 to 40.8)
Body mass index	Family history of breast cancer	11.8 (10.3 to 13.0)	11.8 (10.3 to 13.0)
Body mass index	History of benign breast disease	21.4 (19.5 to 22.7)	21.6 (19.5 to 23.1)
Body mass index	Age at menarche	23.5 (21.8 to 24.6)	26.2 (24.3 to 27.6)
Body mass index	Number of live births	13.4 (12.0 to 14.4)	12.7 (11.3 to 13.8)
Body mass index	Age at first live birth^b^	23.7 (21.6 to 25.1)	23.8 (21.6 to 25.4)
Body mass index	Hormone replacement therapy use	22.9 (21.1 to 24.1)	23.4 (21.4 to 24.8)
Body mass index	Breast density	45.9 (43.7 to 47.6)	43.7 (41.3 to 45.4)
Ethnicity	Family history of breast cancer	56.6 (55.6 to 57.4)	61.0 (59.8 to 61.8)
Ethnicity	History of benign breast disease	49.3 (48.4 to 50.1)	52.5 (51.3 to 53.2)
Ethnicity	Age at menarche	52.5 (51.7 to 53.1)	60.8 (59.9 to 61.4)
Ethnicity	Number of live births	61.1 (60.0 to 62.0)	54.7 (53.3 to 55.8)
Ethnicity	Age at first live birth^b^	51.9 (50.9 to 52.6)	55.7 (54.6 to 56.5)
Ethnicity	Hormone replacement therapy use	52.4 (51.5 to 53.0)	51.4 (50.4 to 52.1)
Ethnicity	Breast density	68.8 (67.3 to 69.9)	67.3 (65.6 to 68.6)
Breast density	Family history of breast cancer	52.1 (49.9 to 53.8)	49.1 (46.3 to 51.0)
Breast density	History of benign breast disease	50.1 (47.9 to 51.8)	46.9 (44.2 to 48.8)
Breast density	Age at menarche	56.5 (54.5 to 58.1)	53.7 (51.3 to 55.4)
Breast density	Number of live births	32.0 (29.8 to 33.7)	29.8 (27.3 to 31.6)
Breast density	Age at first live birth^b^	47.4 (44.8 to 49.3)	44.2 (41.4 to 46.3)
Breast density	Hormone replacement therapy use	48.9 (46.6 to 50.6)	45.8 (43.1 to 47.7)

^a^Categories with undefined odds ratios were not used in the calculation of $\frac{{{pd}}_{j}}{{{RR}}_{j}}$, however they are included in obtaining ${{pd}}_{j}$ of other categories.

^b^Excludes women who are nulliparous.

### PARs of non-modifiable risk factors

Malays (reference level) were found to have the lowest risk of breast cancer, followed by Indians (OR 1.95, 95% CI: 1.00–3.81) and Chinese (OR 2.06, 95% CI: 1.17–3.62) (Table 2). The PAR of ethnicity was 49.4% (95% CI: 48.6–50.0). The proportions of all breast cancer cases attributable to a family history of breast cancer (3.8%, 95% CI: 2.6–4.8) and personal history of benign breast disease (4.3%, 95% CI: 3.2–5.2) were relatively low. Combinations of ethnicity and risk factors (excluding BMI) yielded PAR between 49.3% (history of benign breast disease, 95% CI: 48.4–50.1) and 68.8% (breast density, 95% CI: 67.3–69.9) in all women (Table 3).

### PARs of reproductive and hormonal factors

A smaller proportion of breast cancer cases appeared to be attributable to reproductive and hormonal factors (Table 2). If all women’s age at menarche was aged 14 or older 9.2% (95% CI: 8.2–9.8) of breast cancers could be avoided, 4.2% (95% CI: 3.3–5.0) if all women had at least one child.

### PARs of breast density

Up to 48.6% (95% CI: 46.4–50.3) of all breast cancers can potentially be prevented if all women had breast density ≤12.29% (Table 2). The lowest PAR for combinations of breast density with non-modifiable risk or reproductive and hormonal factors was 32.0% (number of live births, 95% CI: 29.8–33.7) in all women. The majority of our findings were not appreciably different in a subset of post-menopausal women (Tables 2 and 3). The PAR estimates in Chinese women were similar to those of all women (Supplementary Tables 4 and 5).

### Absolute risks based on risk categories of non-modifiable risk factors

**Figure 2:**
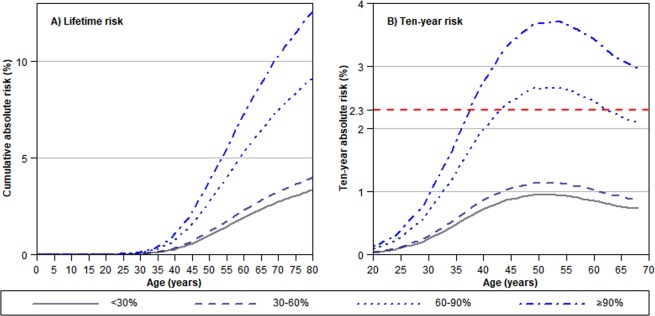
Cumulative lifetime and ten-year absolute risks for developing breast cancer for women in Singapore. Presented by percentiles of risk from non-modifiable risk factors (ethnicity, family history of breast cancer, history of benign breast disease, age at menarche, number of live births, and age at first live birth). Absolute risk was computed only for non-modifiable risk factors as the values do not change over a woman’s lifetime. The intersection of the different risk curves with the red dashed line in (**B**) indicates the age at which women in different risk categories would reach the same ten-year absolute risk (2.3%) of women who start screening at age 50 according to Surveillance, Epidemiology, and End Results (SEER) statistics^33^.

The median discriminatory accuracy, measured by AUC, of non-modifiable risk factors on predicting breast cancer risk is 62.9% (interquartile range: 61.7–64.1). Women with the highest risk of breast cancer (>90^th^ percentile) as categorized by non-modifiable risk factors were 1.98 (95% CI: 1.73–2.27) times more likely to develop breast cancer than women with average risks (30–60^th^ percentile) (Table 4). The lifetime risk of developing breast cancer for women in the bottom 30% and top 10% of the risk distribution is 3.1% and 10.2%, respectively (Fig. 2A). The absolute risk of developing breast cancer in the next 10 years for women at age 50, the widely recommended age to start biennial mammography screening, is 2.3%^33^. In our cohort the absolute risk at age 50 was 1.8% for Chinese, 0.8% for Malays, and 2.1% for Indians. Comparing the highest risk category (>90^th^ percentile and above) to the lowest risk category (30^th^ percentile and below), more than two times as many breast cancer cases were found. Women in the bottom 60% of the risk distribution will never reach the risk threshold for screening, while women in the highest risk group will reach the risk level recommended for mammography screening when they are ~41 years old (Fig. 2B).

**Table 4:** Percentiles and odds ratio used in Fig. 2.

Percentile of predicted risk (%)^a^	OR (95% CI)^b^	Lifetime risk (%)	10-year risk at 50 years of age (%)	Age at which 10-year risk ≥2.3%
<30	0.60 (0.51–0.69)	3.1	0.9	—
30–60	1.00 (Reference)	5.1	1.5	—
60–90	1.74 (1.56–1.94)	8.9	2.6	44
≥90	1.98 (1.73–2.27)	10.2	3.0	41

^a^Logistic model (built using the training dataset) of the association of breast cancer and non-modifiable risk factors (ethnicity, family history of breast cancer, history of benign breast disease, age at menarche, and age at first live birth). Cut-off of predicted risk is obtained using the testing dataset. ^b^Odds ratios are obtained using the bootstrap method (2000 iterations) on the testing dataset.

CI: Confidence interval, OR: Odds ratio.

The inclusion of modifiable risk factors (BMI and smoke) and breast density resulted in slight improvement in the median discriminatory accuracy (AUC [interquartile range]: 65.6 [64.5–66.8]). The risk model was improved in its ability to differentiate women in the 60–100 percentile from women in the 0–60 percentile (Supplementary Fig. 1). The highest risk group (90–100 percentile) would reach the ten-year risk of 2.3% at age ~38 years, ~3 years earlier than the age estimated using only non-modifiable risk factors. (Supplementary Table 6).

## Discussion

The modifiable risk factor, BMI, has the potential to reduce up to 16.2% of all breast cancer cases in Singapore if all women with high BMI in the population were to attain a BMI of <25 kg/m^2^. High breast density, a strong risk factor for breast cancer, and ethnicity had PAR of close to 50% for breast cancer in Singapore. Emerging evidence has shown that prophylactic treatment with drugs such as Tamoxifen can induce a reduction in breast density, which is in turn linked to decreased breast cancer risk^34–36^. Hence, we also examined breast density as a potentially modifiable risk factor. Other studied breast cancer risk factors (smoking, family history of breast cancer, history of benign breast disease, age at menarche, number of live births, age at first live birth, ever breastfeed, contraceptive use, and HRT use) were individually associated with breast cancer PAR of no more than 10%. Sixty percent of the women with breast cancer risk categorized by non-modifiable risk factors will never reach the risk threshold recommended for mammography screening.

The PAR (17.7%) of BMI in our study falls in the range of 2.4%^13^ to 22.8%^8^ observed in post-menopausal Western women. Excess adipose tissue and increased aromatase activity in obese post-menopausal women may increase their levels of circulating endogenous estrogen, which in turn increases breast cancer risk^37–42^. This finding has potential repercussions for public health as BMI is one of the risk factors that are easily measurable and modifiable.

Concordant with previous studies, we found that Malay women were less likely to develop breast cancer^43,44^. Possible reasons reported include the tendency of Malay women to have more children, have their first child at a younger age and breastfeed over longer periods^44^. However, after adjusting for these factors, PAR of ethnicity for breast cancer remained large, 49.3% in all women and 52.4% in post-menopausal women. Additional socio-cultural factors, such as dietary preferences influenced by their religious beliefs may also play a role^43^.

Unlike other risk factors, the high PAR of ethnicity is mainly due to the much larger proportion of Chinese than Malays (i.e. ~80% of the population will have a reduced risk of breast cancer if the risk of breast cancer in Chinese is reduced to the level of Malays). Similarly, the risk associated with breast cancer is high in women with a family history of breast cancer (OR = 2.31). However, few women reported a family history of breast cancer, resulting in fewer potentially avertable breast cancers when family history is considered a risk factor.

Less than 10% of breast cancers in Singapore were attributable to the young age at menarche (age ≤13) after we accounted for other factors like breast density and ethnicity. This is consistent with findings by Tamimi *et al*.^9^ and Barnes *et al*.^13^, who reported PAR of 8.6% and 7.7% respectively. Sprague *et al*. reported a higher PAR estimate of 18.8%, using women who were at least 15 years at menarche as the reference group^11^. The association between early menarche and increased breast cancer risk can be attributed to the earlier exposure and higher levels of estrogen experienced by women who had early menarche^45^. Similar to the findings by Li *et al*.^20^ and Park *et al*.^18^ PARs for reproductive factors was small.

Our study in is agreement with results from Western populations that, among the well-known risk factors of breast cancer, breast cancer is highly attributable to high breast density^8^. The association between breast density and breast cancer is well-established and confirmed by many studies since it was first described by Wolfe in 1976^46,47^. Women with dense breasts were reported to have a four to six times greater risk of breast cancer compared to those without any visible density, with 26–28% of all breast cancer cases being attributable to having breast densities greater than 50%^48,49^. While reducing breast density holds higher potential in decreasing the risk of breast cancer as compared to lowering BMI, the use of anti-estrogen drugs such as Tamoxifen to reduce breast density is not common practice^34–36^. Obesity is related to a multitude of other diseases such as type 2 diabetes and coronary heart disease^50^. In view of a high prevalence of sedentary behavior and general lack of physical activity, much can be achieved to improve overall health and decrease breast cancer incidence by lowering BMI^51^.

Risk factors such as ethnicity, family history of breast cancer, personal history of benign breast disease, age at menarche, and number live births are by nature non-modifiable. Information on non-modifiable risk factors can help reduce breast cancer incidence by means of stratifying the population at risk of developing breast cancer for targeted screening. The current nationwide screening strategy,which started in year 2002, recommends that women aged 40–49 years go for routine mammography screening every year, and women aged 50 years and above, every two years^17^. However, only 66% of the main target group of women aged 50 to 69 ever had a mammogram, and half of them do not come back for regular screening at two-year intervals, negating the benefit of mammography (Health Promotion Board, Singapore). Conveying how easily assessable non-modifiable risk factors can affect the risk of breast cancer may persuade high risk women to go for regular screening.

We acknowledge that our study has some limitations. Our screening population only included women who were at least 50 years of age, of whom ~90% were post-menopausal. As such, our results may not be generalizable to pre-menopausal women and women of younger age. Due to the small numbers of Malay and Indian participants, we were not able to estimate PAR for each risk factors by ethnicity. However, the impact of the lack of ethnicity specific estimates may not be large in Singapore (74.3% of the population is Chinese and 13.4% Malays^21^). Participation in opportunistic screening during the study period between 1992–1994 was low as mammography screening was expensive^52^. Women attending screening may also be more health conscious, which can potentially underestimate PAR. The sample size was also limited; thus, we could only examine broader absolute risk categories. In the consideration of breast density as a modifiable risk factor, the drugs used to induce breast density reduction may also reduce breast cancer risk through other mechanisms. In this case, the PAR associated with breast density may be overestimated^34^. In addition, the associations between some risk factors and breast cancer may differ according to the tumor subtype^13,53^. For example, Millikan *et al*.^53^ found that basal-like cases exhibited opposite associations to those observed for luminal A for risk factors including parity, age at first pregnancy and breastfeeding. Further stratification by tumor subtype may more clearly reveal the relative importance of risk factors for each subtype.

## Conclusion

A substantial proportion of breast cancers (16.2%) in SBCSP can be attributed to overweight/obesity. In addition, up to 45.9% of breast cancer could potentially be avoided if all women had low BMI and breast density. Women with breast cancer risk below the 60^th^ percentile as categorized by easily assessable non-modifiable risk factors—ethnicity, family history of breast cancer, history of benign breast disease, age at menarche, number of live births, and age at first live birth—will never reach the risk level recommended for screening. Risk-stratification strategies to increasing screening in women with high risk may be a potential strategy to improve breast screening guidelines. Understanding the attributable risk of known risk factors on breast cancer incidence may motivate health policy changes, possibly through personalized risk-reduction by introducing target intervention such as Tamoxifen (as a prevention) to reduce their risk.

## Supplementary information

Supplementary Information.

## Data Availability

The data underlying the results presented in the study are third party data and are not publicly available. The data is available by request from Ms Tan Hui Shan, tan.hui.shan@nccs.com.sg.
