# Supplementary material for: Incidence of breast cancer attributable to breast density, modifiable and non-modifiable breast cancer risk factors in Singapore
Source: Sci Rep. 2020 Jan 16;10:503. doi: 10.1038/s41598-019-57341-7 (PMC6965174; doi:10.1038/s41598-019-57341-7)

**Incidence of breast cancer attributable to breast density, modifiable and non-modifiable breast cancer risk factors**

**Running title: Breast cancer attributable risk factors**

Peh Joo Ho^1,2^ ho_peh_joo@gis.a-star.edu.sg

Hannah Lau Si Hui^1,3^ [lauhannah@gmail.com](mailto:lauhannah@gmail.com)

Weang Kee Ho^4,5^ [WeangKee.Ho@nottingham.edu.my](mailto:WeangKee.Ho@nottingham.edu.my)

Fuh Yong Wong^6^ [wong.fuh.yong@singhealth.com.sg](mailto:wong.fuh.yong@singhealth.com.sg)

Qian Yang^2^ yang_qian@nus.edu.sg

Ken Wei Tan^2^  kenwei@nus.edu.sg

Min-Han Tan^6,7^  [mhtan@ibn.a-star.edu.sg](mailto:mhtan@ibn.a-star.edu.sg)

Wen Yee Chay^6^ [chay.wen.yee@singhealth.com.sg](mailto:chay.wen.yee@singhealth.com.sg)

Kee Seng Chia^2^ kee_seng_chia@nus.edu.sg

Mikael Hartman^2,8^ ephbamh@nus.edu.sg

^*^Jingmei Li^1,8^ lijm1@gis.a-star.edu.sg

^1^ Genome Institute of Singapore, 60 Biopolis Street, Genome, #02-01, Singapore 138672, Singapore

^2^ Saw Swee Hock School of Public Health, National University of Singapore and National University Health System, Singapore

^3^ Faculty of Science, National University of Singapore, Singapore

^4^ Department of Applied Mathematics, Faculty of Engineering, University of Nottingham Malaysia, Malaysia

^5^ Cancer Research Malaysia, 1 Jalan SS12/1A, Subang Jaya, 47500 Selangor, Malaysia

^6^ National Cancer Centre Singapore, Singapore

^7^ Institute of Bioengineering and Nanotechnology, Singapore

^8^ Department of Surgery, Yong Loo Lin School of Medicine National University of Singapore

***Correspondence to:** Jingmei Li, Genome Institute of Singapore, 60 Biopolis Street, Genome, #02-01, Singapore 138672, Singapore. Tel: (65) 6808 8312; Email: [lijm1@gis.a-star.edu.sg](mailto:lijm1@gis.a-star.edu.sg)

**Supplementary Table 1**: Mean and variance of continuous variables (percent density, BMI, age at menarche, and age at first birth) by age group in years.

|  | **Sample size** | **Mean** | **Variance** |
| --- | --- | --- | --- |
| **Percentage breast density** |  |  |  |
| **Age group, years** |  |  |  |
| 50 – 54 | 7,374 | 24.32 | 117.65 |
| 55 – 59 | 9,040 | 20.27 | 100.11 |
| 60 – 64 | 6,465 | 16.48 | 79.56 |
| 65 – 69 | 1,486 | 14.55 | 72.99 |
| **Body mass index** |  |  |  |
| **Age group, years** |  |  |  |
| 50 – 54 | 8,386 | 24.55 | 16.93 |
| 55 – 59 | 10,496 | 24.64 | 17.11 |
| 60 – 64 | 7,497 | 25.02 | 18.30 |
| 65 – 69 | 1,740 | 25.05 | 16.43 |
| **Age at menarche, years** |  |  |  |
| **Age group, years** |  |  |  |
| 50 – 54 | 8,385 | 14.03 | 3.18 |
| 55 – 59 | 10,491 | 14.31 | 3.45 |
| 60 – 64 | 7,500 | 14.85 | 3.72 |
| 65 – 69 | 1,742 | 15.11 | 4.40 |
| **Age at first live birth, years, in women with at least one live birth** | | | |
| **Age group, years** |  |  |  |
| 50 – 54 | 7,623 | 24.56 | 22.98 |
| 55 – 59 | 9,627 | 23.96 | 23.65 |
| 60 – 64 | 7,014 | 23.07 | 22.95 |
| 65 – 69 | 1,644 | 22.34 | 19.82 |

**Supplementary Table 2**: Covariance between continuous variables (percent density, BMI, age at menarche, and age at first birth) by age group in years.

|  | **Body mass index** | **Age at menarche, years** | **Age at first live birth, years** |
| --- | --- | --- | --- |
| **Age group 50 – 54 years** |  |  |  |
| **Percentage breast density** | -14.53 | 0.12 | 10.14^1^ |
| **Body mass index** | - | -0.75 | -3.81^1^ |
| **Age at menarche, years** | - | - | 0.37^1^ |
| **Age group 55 – 59 years** |  |  |  |
| **Percentage breast density** | -14.78 | 0.05 | 10.69^1^ |
| **Body mass index** | - | -0.82 | -4.15^1^ |
| **Age at menarche, years** | - | - | 0.30^1^ |
| **Age group 60 – 64 years** |  |  |  |
| **Percentage breast density** | -12.01 | -0.15 | 8.88^1^ |
| **Body mass index** | - | -0.83 | -3.15^1^ |
| **Age at menarche, years** | - | - | 0.55^1^ |
| **Age group 65 – 69 years** |  |  |  |
| **Percentage breast density** | -11.53 | 0.04 | 9.38 |
| **Body mass index** | - | -1.28 | -2.54 |
| **Age at menarche, years** | - | - | 0.72 |

^1^Excludes women who are nulliparous.

**Supplementary Table 3:** Distribution of modifiable, non-modifiable, reproductive and hormonal risk factors, and breast density in 28,130 Asian women, by age group.

|  | **Age group, years** | | | |  |
| --- | --- | --- | --- | --- | --- |
|  | **50 – 54** | **55 – 59** | **60 – 64** | **65 – 69** |  |
|  | ***n*=8,389 (29.8)** | ***n*=10,498 (37.3)** | ***n*=7,500 (26.7)** | ***n*=1,743 (6.2)** | **P-value** |
| **Modifiable** |  |  |  |  |  |
| **Body mass index group (kg/m^2^)** |  |  |  |  |  |
| < 18.5 | 4,604 (54.9) | 5,484 (52.2) | 3,687 (49.2) | 860 (49.3) | <0.001 |
| 18.5 – 24.9 | 372 (4.4) | 512 (4.9) | 317 (4.2) | 70 (4.0) |  |
| 25.0 – 29.9 | 2,603 (31.0) | 3,512 (33.5) | 2,627 (35) | 626 (35.9) |  |
| 30.0 – 34.9 | 668 (8.0) | 799 (7.6) | 721 (9.6) | 152 (8.7) |  |
| ≥ 35.0 | 139 (1.7) | 189 (1.8) | 145 (1.9) | 32 (1.8) |  |
| Unknown | 3 (0.0) | 2 (0.0) | 3 (0.0) | 3 (0.2) |  |
|  |  |  |  |  |  |
| **Smoke (Ever)** |  |  |  |  |  |
| No | 8,069 (96.2) | 9,896 (94.3) | 6,862 (91.5) | 1,558 (89.4) | <0.001 |
| Yes | 320 (3.8) | 602 (5.7) | 638 (8.5) | 185 (10.6) |  |
|  |  |  |  |  |  |
| **Non-modifiable** |  |  |  |  |  |
| **Ethnicity** |  |  |  |  |  |
| Chinese | 7,078 (84.4) | 8,894 (84.7) | 6,238 (83.2) | 1,472 (84.5) | 0.036 |
| Malay | 458 (5.5) | 538 (5.1) | 485 (6.5) | 101 (5.8) |  |
| Indian | 411 (4.9) | 527 (5.0) | 382 (5.1) | 76 (4.4) |  |
| Other | 442 (5.3) | 539 (5.1) | 395 (5.3) | 94 (5.4) |  |
|  |  |  |  |  |  |
| **Family history of breast cancer** |  |  |  |  |  |
| No | 8,053 (96.0) | 10,103 (96.2) | 7,249 (96.7) | 1,669 (95.8) | 0.02 |
| Yes | 252 (3.0) | 268 (2.6) | 168 (2.2) | 40 (2.3) |  |
| Unknown | 84 (1.0) | 127 (1.2) | 83 (1.1) | 34 (2.0) |  |
|  |  |  |  |  |  |
| **History of benign breast disease** |  |  |  |  |  |
| No | 7,859 (93.7) | 9,943 (94.7) | 7,165 (95.5) | 1,685 (96.7) | <0.001 |
| Yes | 530 (6.3) | 555 (5.3) | 335 (4.5) | 58 (3.3) |  |
|  |  |  |  |  |  |
| **Reproductive and hormonal** |  |  |  |  |  |
| **Age at menarche, years** |  |  |  |  |  |
| ≤ 13 | 3,606 (43.0) | 3,875 (36.9) | 1,972 (26.3) | 415 (23.8) | <0.001 |
| 14 – 15 | 3,079 (36.7) | 3,948 (37.6) | 2,909 (38.8) | 612 (35.1) |  |
| ≥ 16 | 1,700 (20.3) | 2,668 (25.4) | 2,619 (34.9) | 715 (41.0) |  |
|  |  |  |  |  |  |
| **Menopausal status** |  |  |  |  |  |
| Pre | 2,431 (29.0) | 464 (4.4) | 24 (0.3) | 4 (0.2) | <0.001 |
| Post | 5,958 (71.0) | 10,034 (95.6) | 7,476 (99.7) | 1,739 (99.8) |  |
|  |  |  |  |  |  |
| **Number of live births** |  |  |  |  |  |
| 0 | 711 (8.5) | 789 (7.5) | 450 (6.0) | 95 (5.5) | <0.001 |
| 1-2 | 2,361 (28.1) | 4,299 (41.0) | 4,298 (57.3) | 1,126 (64.6) |  |
| 3-4 | 1,698 (20.2) | 1,638 (15.6) | 865 (11.5) | 173 (9.9) |  |
| ≥5 | 3,619 (43.1) | 3,772 (35.9) | 1,887 (25.2) | 349 (20.0) |  |
|  |  |  |  |  |  |
| **Age at first live birth^1^** |  |  |  |  |  |
| Age ≤ 30 years | 6,917 (90.1) | 8,827 (90.9) | 6,515 (92.4) | 1,566 (95.0) | <0.001 |
| Age > 30 years | 761 (9.9) | 882 (9.1) | 535 (7.6) | 82 (5.0) |  |
|  |  |  |  |  |  |
| **Ever breastfed^1^** |  |  |  |  |  |
| No | 2,703 (35.2) | 3,198 (32.9) | 1,733 (24.6) | 337 (20.4) | <0.001 |
| Yes | 4,920 (64.1) | 6,429 (66.2) | 5,281 (74.9) | 1,307 (79.3) |  |
| Unknown | 55 (0.7) | 82 (0.8) | 36 (0.5) | 4 (0.2) |  |
|  |  |  |  |  |  |
| **Contraceptive** |  |  |  |  |  |
| No | 4,516 (53.8) | 6,318 (60.2) | 5,199 (69.3) | 1,350 (77.5) | <0.001 |
| Yes | 3,873 (46.2) | 4,180 (39.8) | 2,301 (30.7) | 393 (22.5) |  |
|  |  |  |  |  |  |
| **HRT use** |  |  |  |  |  |
| No | 6,865 (81.8) | 8,978 (85.5) | 6,908 (92.1) | 1,627 (93.3) | <0.001 |
| Yes | 1,524 (18.2) | 1,520 (14.5) | 592 (7.9) | 116 (6.7) |  |
|  |  |  |  |  |  |
| **Others** |  |  |  |  |  |
| **Breast density** |  |  |  |  |  |
| 0 – 12.29 | 909 (10.8) | 2,058 (19.6) | 2,379 (31.7) | 690 (39.6) | <0.001 |
| 12.30 – 18.24 | 1,448 (17.3) | 2,301 (21.9) | 1,902 (25.4) | 413 (23.7) |  |
| 18.25 – 26.03 | 2,143 (25.5) | 2,426 (23.1) | 1,291 (17.2) | 246 (14.1) |  |
| 26.04 – 100 | 2,874 (34.3) | 2,255 (21.5) | 893 (11.9) | 137 (7.9) |  |
| Unknown | 1,015 (12.1) | 1,458 (13.9) | 1,035 (13.8) | 257 (14.7) |  |

^1^ Excludes women who are nulliparous.

**Supplementary Table 4:** Population attributable risk proportion of known risk factors in 23,692 Chinese women of which 21,157 were post-menopausal. The association of known risk factors and breast cancer was estimated using logistic regression.

|  | **Breast cancer in Chinese women (*n* = 23,692)** | | | | **Breast cancer in post-menopausal women (*n* = 21,157)** | | | |
| --- | --- | --- | --- | --- | --- | --- | --- | --- |
|  | **Univariable** | | **Multivariable^1^** | | **Univariable** | | **Multivariable^1^** | |
| **Characteristics** | **OR (95% CI)** | **PAR (95% CI)** | **OR (95% CI)** | **PAR (95% CI)** | **OR (95% CI)** | **PAR (95% CI)** | **OR (95% CI)** | **PAR (95% CI)** |
| **Modifiable** |  |  |  |  |  |  |  |  |
| **BMI (kg/m^2^)** |  |  |  |  |  |  |  |  |
| 18.5 – 24.9 | 1.00 (Reference) | - | 1.00 (Reference) | 16.0 (14.1 to 17.3) | 1.00 (Reference) | 10.7 (10.1 to 11.4) | 1.00 (Reference) | 18.5 (17.7 to 19.8) |
| < 18.5 | 0.61 (0.34 to 1.1) |  | 0.46 (0.25 to 0.82) |  | 0.70 (0.39 to 1.27) |  | 0.52 (0.28 to 0.94) |  |
| 25.0 – 29.9 | 1.15 (0.93 to 1.42) |  | 1.45 (1.16 to 1.81) |  | 1.22 (0.97 to 1.53) |  | 1.53 (1.21 to 1.94) |  |
| 30.0 – 34.9 | 1.47 (1.02 to 2.11) |  | 2.09 (1.44 to 3.03) |  | 1.63 (1.12 to 2.37) |  | 2.32 (1.58 to 3.42) |  |
| ≥ 35.0 | 1.71 (0.75 to 3.89) |  | 2.47 (1.08 to 5.68) |  | 1.99 (0.87 to 4.55) |  | 2.86 (1.24 to 6.59) |  |
|  |  |  |  |  |  |  |  |  |
| **Smoke** |  |  |  |  |  |  |  |  |
| No | 1.00 (Reference) | - | 1.00 (Reference) | - | 1.00 (Reference) | - | 1.00 (Reference) | - |
| Yes | 0.71 (0.45 to 1.12) |  | 0.92 (0.60 to 1.40) |  | 0.92 (0.60 to 1.40) |  | 0.92 (0.60 to 1.40) |  |
|  |  |  |  |  |  |  |  |  |
| **Non-modifiable** |  |  |  |  |  |  |  |  |
| **Age group, years** |  |  |  |  |  |  |  |  |
| 50-69 | 1.00 (Reference) | - | 1.00 (Reference) | - | 1.00 (Reference) | - | 1.00 (Reference) | - |
| ≥ 70 | 0.79 (0.63 to 0.98) |  | 1.14 (0.90 to 1.44) |  | 0.81 (0.65 to 1.02) |  | 1.12 (0.88 to 1.43) |  |
|  |  |  |  |  |  |  |  |  |
|  |  |  |  |  |  |  |  |  |
| **Family history of breast cancer** |  |  |  |  |  |  |  |  |
| No | 1.00 (Reference) | 3.2 (1.9 to 4.2) | 1.00 (Reference) | 2.7 (1.6 to 3.5) | 1.00 (Reference) | 3.5 (3.1 to 3.7) | 1.00 (Reference) | 3.1 (2.7 to 3.3) |
| Yes | 2.29 (1.49 to 3.52) |  | 1.92 (1.24 to 2.96) |  | 2.49 (1.59 to 3.9) |  | 2.09 (1.32 to 3.29) |  |
| Unknown | 0.21 (0.03 to 1.47) |  | 0.18 (0.02 to 1.26) |  | - |  | - |  |
|  |  |  |  |  |  |  |  |  |
| **History of benign breast disease** |  |  |  |  |  |  |  |  |
| No | 1.00 (Reference) | 5.1 (3.8 to 6.3) | 1.00 (Reference) | 4.0 (2.9 to 4.8) | 1.00 (Reference) | 4.8 (4.8 to 5.3) | 1.00 (Reference) | 3.8 (3.7 to 4.2) |
| Yes | 2.00 (1.44 to 2.76) |  | 1.63 (1.17 to 2.26) |  | 1.97 (1.38 to 2.81) |  | 1.63 (1.14 to 2.34) |  |
|  |  |  |  |  |  |  |  |  |
| **Reproductive and hormonal** |  |  |  |  |  |  |  |  |
| **Age at menarche, years ^2^** |  |  |  |  |  |  |  |  |
| 14 – 15 | 1.00 (Reference) | 10.6 (9.4 to 11.6) | 1.00 (Reference) | 8.0 (7.1 to 8.7) | 1.00 (Reference) | 11.0 (9.5 to 12.8) | 1.00 (Reference) | 8.5 (7.3 to 9.9) |
| ≥ 16 | 0.82 (0.63 to 1.06) |  | 0.91 (0.70 to 1.18) |  | 0.88 (0.67 to 1.15) |  | 0.98 (0.74 to 1.29) |  |
| ≤ 13 | 1.36 (1.09 to 1.7) |  | 1.25 (0.99 to 1.57) |  | 1.39 (1.09 to 1.77) |  | 1.28 (1.00 to 1.63) |  |
|  |  |  |  |  |  |  |  |  |
| **Number of live births** |  |  |  |  |  |  |  |  |
| 1-2 | 1.00 (Reference) | 4.7 (3.7 to 5.6) | 1.00 (Reference) | - | 1.00 (Reference) | 4.7 (4.7 to 5.0) | 1.00 (Reference) | - |
| 0 | 1.53 (1.11 to 2.11) |  | 1.39 (0.98 to 1.97) |  | 1.53 (1.11 to 2.11) |  | 1.33 (0.92 to 1.92) |  |
| 3-4 | 0.88 (0.68 to 1.14) |  | 0.95 (0.73 to 1.25) |  | 0.88 (0.68 to 1.14) |  | 0.89 (0.66 to 1.19) |  |
| ≥5 | 0.54 (0.41 to 0.7) |  | 0.66 (0.49 to 0.90) |  | 0.54 (0.41 to 0.7) |  | 0.63 (0.46 to 0.87) |  |
|  |  |  |  |  |  |  |  |  |
| **Age at first live birth ^2^** |  |  |  |  |  |  |  |  |
| Age ≤ 30 years | 1.00 (Reference) | 4.4 (3.2 to 5.3) | 1.00 (Reference) | - | 1.00 (Reference) | 4.2 (4.3 to 5.2) | 1.00 (Reference) | - |
| Age > 30 years | 1.48 (1.09 to 2.02) |  | 1.14 (0.82 to 1.59) |  | 1.46 (1.05 to 2.04) |  | 1.07 (0.75 to 1.54) |  |
|  |  |  |  |  |  |  |  |  |
| **Ever breastfed ^2^** |  |  |  |  |  |  |  |  |
| Yes | 1.00 (Reference) | 12.9 (11.4 to 14.1) | 1.00 (Reference) | - | 1.00 (Reference) | 13.2 (13.1 to 14.3) | 1.00 (Reference) | - |
| No | 1.44 (1.16 to 1.78) |  | 1.15 (0.91 to 1.45) |  | 1.46 (1.16 to 1.83) |  | 1.15 (0.90 to 1.47) |  |
| Unknown | 0.95 (0.23 to 3.87) |  | 0.69 (0.17 to 2.85) |  | 0.56 (0.08 to 4.05) |  | 0.37 (0.05 to 2.70) |  |
|  |  |  |  |  |  |  |  |  |
| **Contraceptive** |  |  |  |  |  |  |  |  |
| Yes | 1.00 (Reference) | - | 1.00 (Reference) | - | 1.00 (Reference) | 14.3 (13.6 to 15.2) | 1.00 (Reference) | - |
| No | 1.18 (0.96 to 1.45) |  | 1.06 (0.85 to 1.32) |  | 1.27 (1.01 to 1.58) |  | 1.13 (0.89 to 1.43) |  |
|  |  |  |  |  |  |  |  |  |
| **HRT use** |  |  |  |  |  |  |  |  |
| No | 1.00 (Reference) | 7.2 (5.8 to 8.2) | 1.00 (Reference) | - | 1.00 (Reference) | 7.1 (6.2 to 8.0) | 1.00 (Reference) | - |
| Yes | 1.54 (1.21 to 1.96) |  | 1.27 (0.99 to 1.63) |  | 1.57 (1.20 to 2.04) |  | 1.26 (0.96 to 1.66) |  |
|  |  |  |  |  |  |  |  |  |
| **Others** |  |  |  |  |  |  |  |  |
| **Breast density** |  |  |  |  |  |  |  |  |
| 0 – 12.29 | 1.00 (Reference) | 46.8 (44.4 to 48.5) | 1.00 (Reference) | 49.9 (47.4 to 51.7) | 1.00 (Reference) | 44.0 (43.3 to 46.0) | 1.00 (Reference) | 47.4 (46.7 to 49.5) |
| 12.30 – 18.24 | 1.46 (0.99 to 2.14) |  | 1.53 (1.04 to 2.27) |  | 1.42 (0.96 to 2.1) |  | 1.52 (1.02 to 2.25) |  |
| 18.25 – 26.03 | 2.19 (1.53 to 3.13) |  | 2.40 (1.65 to 3.49) |  | 2.17 (1.51 to 3.12) |  | 2.42 (1.66 to 3.54) |  |
| 26.04 – 100 | 3.11 (2.21 to 4.38) |  | 3.46 (2.38 to 5.03) |  | 3.00 (2.11 to 4.26) |  | 3.36 (2.28 to 4.95) |  |
| Unknown | 1.10 (0.69 to 1.76) |  | 1.12 (0.70 to 1.80) |  | 0.94 (0.57 to 1.56) |  | 0.96 (0.58 to 1.60) |  |

**Supplementary Table 5:** Population attributable risk (PAR) for combinations of risk factors in 21,157 Chinese women. Where the risk factors (as categorical variables) were not studied in combination, the following risk factors were adjusted for breast density (as a continuous variable), body mass index (as a continuous variable), ethnicity, age at recruitment (as a continuous variable), family history of breast cancer, age at menarche (as a continuous variable), age at first live birth, and hormone replacement therapy use.

|  |  | **Population attributable risk (95% Confidence Interval)^1^** | |
| --- | --- | --- | --- |
| **Risk factor 1** | **Risk factor 2** | **Chinese women** | **Post-menopausal Chinese women** |
| **Body mass index** | **Family history of breast cancer** | 21.0 (18.7 to 22.6) | 23.4 (5.5 to 25.0) |
| **Body mass index** | **History of benign breast disease** | 21.7 (19.6 to 23.1) | 22.7 (20.3 to 24.6) |
| **Body mass index** | **Age at menarche** | 23.3 (21.4 to 24.6) | 26.6 (24.4 to 28.1) |
| **Body mass index** | **Number of live births** | 14.8 (13.3 to 15.9) | 13.8 (12.0 to 15.1) |
| **Body mass index** | **Age at first live birth^2^** | 24.3 (22.1 to 25.9) | 25.6 (23.0 to 27.5) |
| **Body mass index** | **Hormone replacement therapy use** | 23.3 (21.2 to 24.8) | 24.8 (22.5 to 26.6) |
| **Body mass index** | **Breast density** | 49.9 (47.4 to 51.8) | 47.5 (44.8 to 49.5) |
| **Breast density** | **Family history of breast cancer** | 51.8 (49.4 to 53.6) | 49.4 (46.7 to 51.5) |
| **Breast density** | **History of benign breast disease** | 51.8 (49.3 to 53.6) | 49.1 (46.2 to 51.1) |
| **Breast density** | **Age at menarche** | 59.8 (57.6 to 61.4) | 57.4 (54.9 to 59.2) |
| **Breast density** | **Number of live births** | 29.9 (27.5 to 31.7) | 27.7 (25.2 to 29.5) |
| **Breast density** | **Age at first live birth^2^** | 48.6 (46.0 to 50.6) | 46.2 (43.3 to 48.4) |
| **Breast density** | **Hormone replacement therapy use** | 50.7 (48.2 to 52.6) | 48.3 (45.4 to 50.3) |

^1^ Categories with undefined odds ratios were not used in the calculation of $\frac{{pd}_{j}}{{RR}_{j}}$, however they are included in obtaining ${pd}_{j}$ of other categories.
^2^ Excludes women who are nulliparous.

**Supplementary Table 6: Percentiles and odds ratio used in Supplementary Figure 1.**

| **Percentile of predicted risk (%)^1^** | **OR (95% CI)^2^** | **Lifetime risk (%)** | **10-year risk at 50 years of age (%)** | **Age at which 10-year risk ≥ 2.3%** |
| --- | --- | --- | --- | --- |
| < 30 | 0.84 (0.73–0.97) | 3.3 | 0.9 | - |
| 30 – 60 | 1.00 (Reference) | 4.0 | 1.1 | - |
| 60 – 90 | 2.30 (2.04–2.61) | 9.1 | 2.6 | 44 |
| ≥ 90 | 3.20 (2.75–3.66) | 12.6 | 3.7 | 38 |

^1^ Logistic model (built using the training dataset) of the association of breast cancer and non-modifiable risk factors (ethnicity, family history of breast cancer, history of benign breast disease, age at menarche, and age at first live birth). Cut-off of predicted risk is obtained using the testing dataset.
^2^ Odds ratios are obtained using the bootstrap method (2000 iterations) on the testing dataset.

CI: Confidence interval, OR: Odds ratio.

**Supplementary Figure 1**. Cumulative (lifetime and ten-year) absolute risks for developing breast cancer for women in Singapore. Presented by percentiles of risk from non-modifiable risk factors (ethnicity, family history of breast cancer, history of benign breast disease, age at menarche, number of live births, and age at first live birth), modifiable risk factors (body mass index, smoke) and breast density. The intersection of the different risk curves with the red dashed line in (B) indicates the age at which women in different risk categories would reach the same ten-year absolute risk (2.3%) of women who start screening at age 50 according to Surveillance, Epidemiology, and End Results (SEER) statistics (33).


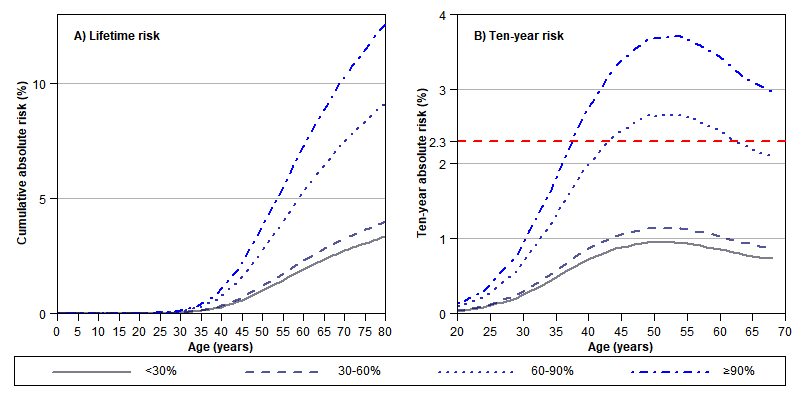

Supplement: Supplementary file 1 — Supplementary Information. [file 41598_2019_57341_MOESM1_ESM.docx]
